# Supplementary material for: Realizing Hydrogen De/Absorption Under Low Temperature for MgH2 by Doping Mn-Based Catalysts
Source: Nanomaterials (Basel). 2020 Sep 3;10(9):1745. doi: 10.3390/nano10091745 (PMC7560042; doi:10.3390/nano10091745)
Supplement: Supplementary file 1 [file nanomaterials-10-01745-s001.pdf]

nanomaterials

Supplementary Materials

Realizing Hydrogen De/Absorption Under Low Temperature for MgH_2 by Doping Mn-Based Catalysts

Ze Sun, Liuting Zhang *, Nianhua Yan, Jiaguang Zheng, Ting Bian, Zongming Yang and Shichuan Su *

College of Energy and Power, Jiangsu University of Science and Technology, Zhenjiang 212003, China; 182210019@stu.just.edu.cn (Z.S.); 189210007@stu.just.edu.cn (N.Y.); 11526056@zju.edu.cn (J.Z.); tingbian89@just.edu.cn (T.B.); Zongmingy@just.edu.cn (Z.Y.)

* Correspondence: zhanglt89@just.edu.cn (L.Z.), jstussc1@163.com (S.S.)

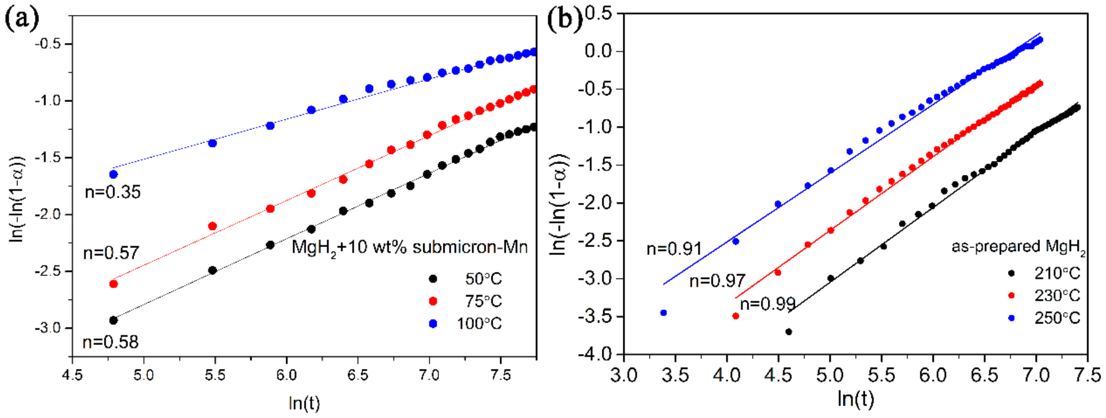

Figure S1. JMAK plots of MgH_2 + 10 wt% submicron-Mn (**a**) composite and MgH_2 (**b**).
